# Metabolomics in the Context of Exercise in Subjects with Multimorbidity: A Pilot Study

**DOI:** 10.3390/biom15101474

**Published:** 2025-10-20

**Authors:** Rebecca Bankamp, Simone Schweda, Nils Janzen, Andreas M. Nieß, Inga Krauß, Barbara Munz

**Affiliations:** 1 Medical Clinic, Department of Sports Medicine, University Hospital Tübingen, Hoppe-Seyler-Str. 6, D-72076 Tübingen, Germanysimone.schweda@med.uni-tuebingen.de (S.S.); andreas.niess@med.uni-tuebingen.de (A.M.N.); inga.krauss@med.uni-tuebingen.de (I.K.); 2 Department of Clinical Chemistry, Hannover Medical School, Carl-Neuberg-Str. 1, D-30625 Hannover, Germany; n.janzen@metabscreen.de; 3 Screening-Labor Hannover, 30430 Hannover, Lower Saxony, Germany, Am Steinweg 11, D-30952 Ronnenberg, Germany; 4 Interfaculty Research Institute for Sport and Physical Activity, Eberhard Karls University of Tübingen, D-72074/72076 Tübingen, Germany

**Keywords:** lifestyle-related diseases, multimorbidity, exercise, metabolomics, dried blood spots

## Abstract

Lifestyle-related diseases, such as overweight/obesity, diabetes mellitus type 2 (T2DM), cardiovascular disease, or osteoarthritis, are a major health burden in Western societies. Due to common risk factors, most patients suffer from multimorbidity, i.e., have been diagnosed with more than one of these diseases. Physical activity (PA) is known to have a positive effect on all of these diseases; however, little is known about the effects of PA on patients with multimorbidity. In particular, so far, no reliable biomarkers have been found to predict and monitor the effects of PA-based lifestyle intervention programs on these subjects. Employing a metabolomics approach with dried blood spots, we analyzed the concentrations of different metabolites in subjects with multimorbidity over the course of the lifestyle intervention program MultiPill-Exercise. We found increased concentrations of all tested amino acids (AAs), total carnitine (Cx), and short- (C2-C6) and long- (>C12) chain acylcarnitines (ACs) after 12 weeks (t1) and/or 24 weeks (t2) of intervention. When correlating baseline (t0) metabolite concentrations with changes in physiological and clinical parameters, we observed associations of various metabolite concentrations with changes in metabolic and cardiovascular parameters. When analyzing metabolite acute reactions in response to exhaustive exercise (ergometer test), however, few overall changes were observed. Nevertheless, a significant negative correlation was found between the mobilization of medium-chain acylcarnitines (MC-ACs) at t2 and changes in peak power output (PPO) between t0 and t2. Taken together, these data suggest that specific AAs and ACs might be candidate biomarkers to predict and monitor the effects of PA-based lifestyle intervention programs in subjects with multimorbidity, a hypothesis that should be further tested in larger cohorts.

## 1. Introduction

Lifestyle-related diseases, specifically overweight/obesity, diabetes mellitus type 2 (T2DM), cardiovascular disease, or osteoarthritis, represent a major health threat in Western societies. Inactivity and an unhealthy diet, excess alcohol consumption, smoking, and psychosocial factors such as stress are important risk factors. Most patients with lifestyle-related disease suffer from multimorbidity, mainly due to the similarity between risk factors for these diseases. Physical exercise is considered “poly-“ or “multipill” in the context of lifestyle-related diseases. While a range of studies clearly demonstrated the preventive, therapeutic, and rehabilitative efficacy of sports and everyday-life physical activity in a broad variety of settings, studies focusing on the aspect of multimorbidity are still scarce (for a review, see [1]).

The “MultiPill-Exercise” study aimed to fill this gap. Organized as a pilot study, followed by a large-scale randomized controlled trial, it focused on enhancing sport and physical activity commitment in subjects with more than one lifestyle-related disease (for details, see [2]). Since a healthier lifestyle is known to yield metabolic benefits, we hypothesized that we might be able to detect changes in subjects’ metabolomes throughout the intervention and that these changes might be associated with individuals’ health benefits, thus allowing metabolic components to be employed as biomarkers for training adaptation and/or tools for individual training management in the future.

The most central players in a person’s metabolome are amino acids (AAs) and acylcarnitines (ACs). Indeed, data from the literature suggest the effects of both acute exercise and exercise training on AA plasma profiles, since AA turnover adapts to physical activity in multiple ways and might reflect metabolic changes in response to sports (for a review, see [3,4,5,6]). Consequently, AAs are interesting candidate biomarkers in the context of exercise, particularly aromatic and branched-chain AAs (BCAAs). Nevertheless, little is known about AA patterns in subjects with multimorbidity. Interestingly, BCAAs, glucagon secretion, and insulin sensitivity are mutually connected in a complex manner, suggesting associations with metabolic syndrome and T2DM pathogenesis (for a review, see [7]). Consistently, data by Hansen et al. [8] suggest differential responses to exercise in people with T2DM with regard to their AA profiles. Similarly, ACs are intermediates of fatty acid metabolism but can also be generated as by-products of BCAA metabolism or derivates of other organic acids [9]. Exercise and training are known to affect both AC and free carnitine concentrations in circulation. Acute exercise is known to enhance concentrations of most ACs, paralleled by decreases in free carnitine levels [4,10]. In addition, several authors demonstrated increased AC concentrations in the context of overweight/obesity, insulin resistance, and T2DM. Their pro-inflammatory activity is associated with decreased fatty acid oxidation and a low level of metabolic flexibility, rendering them important candidate biomarkers in subjects with multimorbidity [11,12]. Interestingly, Huffman et al. [13] demonstrated decreased concentrations of circulating ACs alongside activity-induced improvements in insulin sensitivity. Consistently, data from a large study by the same group suggest that the metabolic signatures of skeletal muscle in subjects at risk of metabolic disease change during a six-month training intervention, and that they are associated with changes in cardiovascular parameters and might be indicators of metabolic flexibility and plasticity [14].

These data suggest that metabolic parameters might be important biomarkers in the context of exercise-based lifestyle intervention programs for subjects with multimorbidity, such as MultiPill-Exercise. For routine applications, however, easy and cost-effective procedures are required, such as an analysis of metabolites from dried spots of capillary blood via tandem mass spectrometry (MS/MS)—a method that has been established for a long time for the detection of metabolic diseases in newborns, specifically in the context of nationwide newborn screening programs [15]—with its major advantage being that it follows highly standardized and strictly process-evaluated analytical pathways. A further advantage, particularly when compared with standard metabolomics analyses using venous blood, is the much easier and less invasive sampling procedure, as well as easier storage and shipment of samples and high degree of cost-effectiveness. We employed this method to detect changes in AA and AC profiles in selected MultiPill-Exercise subjects and correlated patterns with the development of physiological and clinical data throughout the intervention.

## 2. Materials and Methods

### 2.1. MultiPill-Exercise Pilot Study

This study was conducted in accordance with the Declaration of Helsinki, approved by the “Ethics Committee of the Medical Faculty, University of Tübingen” (reference number: 298/2019BO2, 4 June 2019) and registered at the German clinical trial register (DRKS00016702). Written informed consent was obtained from all subjects. Subjects were required to meet “multimorbidity characteristics,” which meant that they had to have been diagnosed with at least two of four lifestyle diseases (T2DM, manifest or risk thereof; overweight/obesity; cardiovascular disease/hypertension; or osteoarthritis (OA)). The lifestyle intervention program was designed as a 24-week schedule, split up into two 12-week blocks, the first of which was more closely supervised and the second more self-directed. The training modalities are depicted in [2]. Briefly, the subjects were advised to undergo 2–3 sessions of endurance training per week, with a total duration of at least 75–150 min, depending on intensity, in combination with machine-based strength training 2–3 times a week. Functional training, movement teasers, and individual counseling complemented the intervention. Diagnostics, including blood sampling, were performed at baseline (t0), after the first 12-week block (t1) and at the end of the intervention (t2) (for details, see [2]). Spiroergometry was carried out at each of the three diagnostics time points to determine individual cardiorespiratory fitness and was designed as an incremental test on a bike ergometer (for details, see [2]). Altogether, n = 39 subjects were included in this study, who were analyzed in two “waves,” one starting in late August of 2019 (n = 20; MP1901-1925) and the other in mid-January of 2020 (n = 19; MP1927-1953). Missing numbers were assigned to potential subjects that had to be excluded from this study during diagnostics. Due to the COVID-19 pandemic, t1 diagnostics for all subjects of “wave 2” had to be canceled; in addition, for this group, the intervention program had to be significantly modified according to contact restrictions associated with the pandemic (for details, see [2]). Finally, some participants dropped out from this study. As a consequence, “at rest” data for all three time points (t0, t1, and t2) were only available for n = 6 subjects, all of them female, with a mean age of 59.67 years. For subject MP1902 (female, age 54 years), at least t0 and t1 data were available. At t2, metabolomics patterns were also assessed in response to acute exercise; i.e., dried blood spots were sampled before and after spiroergometry diagnostics. Here, data for n = 26 subjects were available.

### 2.2. Metabolomics Analysis from Dried Blood Spots

After disinfection, capillary blood was taken from subjects’ earlobes using a sterile lancet for incision, collected into a lithium heparin glass capillary, and immediately spotted on “Guthrie” paper cards until the designated areas on the cards (denoted by circles) were completely soaked. Subsequently, the cards were allowed to dry in an upright position for 2–4 h, stored at room temperature, and sent to the “Screening Labor Hannover” (https://metabscreen.de/ (accessed on 16 September 2025)) by regular mail. This laboratory is one of the accredited laboratories in Germany for newborn screening, following strict and uniform rules for analytical methodology, process evaluation, and control (for details, see https://www.screening-dgns.de/ (accessed on 16 September 2025)). Analysis was carried out using a flow injection analysis (FIA) (FIA-MS/MS) (for a review, see [16,17,18,19]). For this purpose, the MassScreen^®^ Kit (“amino acids, acylcarnitines, from dried blood, non-derivatized, #57000 including the succinylacetone upgrade set #57111”; Chromsystems, Gräfelfing, Germany) was employed according to the manufacturer’s instructions, giving detailed information on sample preparation, extraction procedures, and MS/MS parameters. Table 1 lists all analyzed metabolites and abbreviations used in this paper. Usually, ACs are classified as short-chained (SC-ACs, C2-C6), medium-chained (MC-ACs, C8-C12), or long-chained (LC-ACs, >C12).

### 2.3. Statistical Analysis

Statistical analysis was carried out using the SPSS software for Macintosh, Version 26.0 (IBM Corporation, Armonk, NY, USA). After compiling all data in a comprehensive table, metabolite baseline concentrations (t0) as well as changes in physiological and clinical parameters after 12 weeks (t0–t1) were tested for normal distribution using the Kolmogorov–Smirnov and Shapiro–Wilk statistical tests. In the case of a normal distribution, t-tests for paired samples were applied; otherwise, Wilcoxon testing for differences was carried out. Potential correlations were tested using Spearman’s rank correlation coefficients. Significance was defined as *p* < 0.05 (*), *p* < 0.01 (**), and *p* < 0.001 (***). The MultiPill-Exercise pilot study represents an exploratory, hypothesis-generating approach. Therefore, no correction factors for multiple testing were introduced.

## 3. Results

### 3.1. Individual Metabolite Kinetics Throughout the Intervention

**Figure 1:**
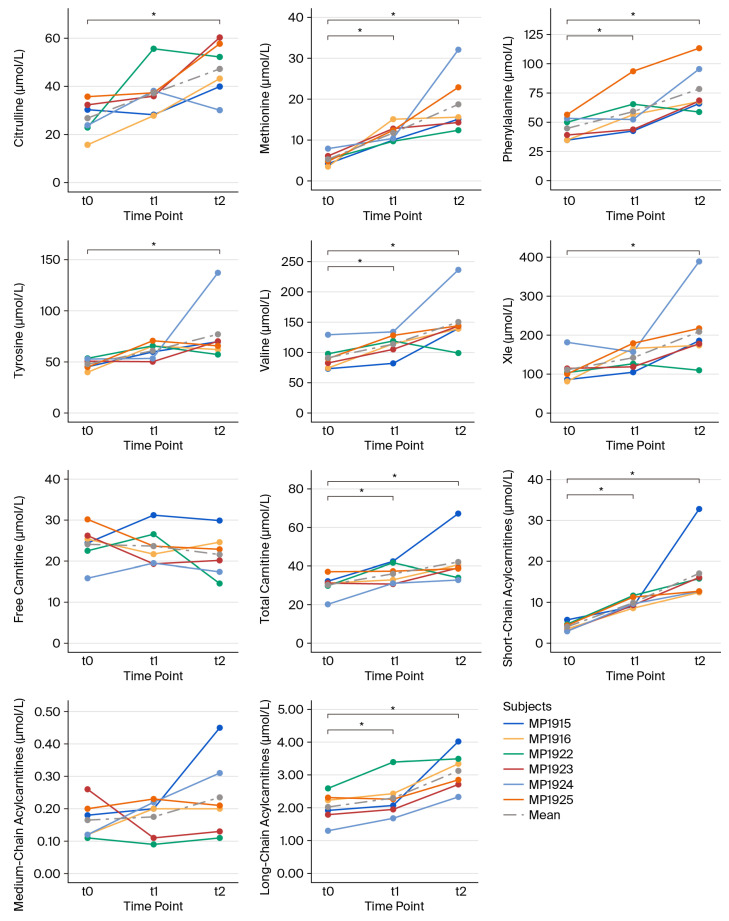
Spaghetti plots of individual metabolite concentrations. Graphs depict changes in individual metabolite concentrations at rest at t0, t1, and t2, as assessed for the six individual subjects for whom data for each time point were available. *: *p* < 0.05.

To characterize individual metabolite patterns, we first determined the time courses for individual subjects throughout the intervention. Unfortunately, for various reasons, mainly technical issues and dropout from the study, complete data sets consisting of t0, t1, and t2 data were only available for n = 6 subjects (MP1915, MP1916, MP1922, MP1923, MP1924, and MP1925); at least t0 and t1 data could be obtained for one additional participant (MP1902); and both t0 and t2 data were available only for n = 21 participants (MP1915, MP1916, MP1922, MP1923, MP1924, MP1925, MP1927, MP1930, MP1932, MP1934, MP1937, MP1940, MP1942, and MP1946-MP1953). As shown in Figure 1 and Table 2, a general increase in all AAs and ACs was found throughout this study, whereas free carnitine showed a moderate decline. In addition, some individual variations were observed, specifically with regard to Cit, Phe, free carnitine, and MC-ACs.

### 3.2. Correlation Analysis

**Figure 2:**
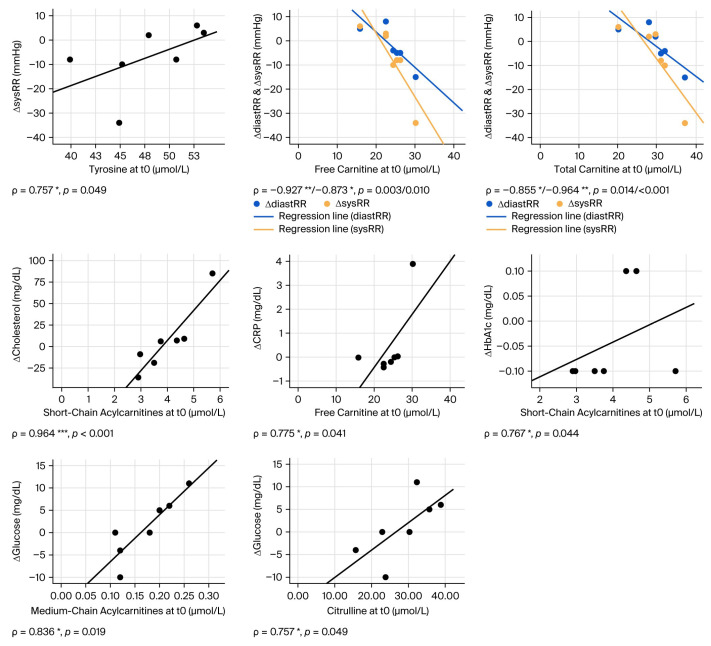
Correlations of baseline metabolite parameters and the development of physiological and clinical parameters between t0 and t1. Graphs depict significant correlations between baseline (t0) metabolite concentrations and changes between different physiological/clinical parameters between t0 and t1, as indicated. *: *p* < 0.05, **: *p* < 0.01, ***: *p* < 0.001.

Against this background, we analyzed potential correlations between metabolite patterns and physiological and clinical parameters. For this purpose, using Spearman’s correlation analysis, we first examined the potential associations between t0 metabolite concentrations and changes in physiological and clinical parameters between t0 and t1, i.e., after 12 weeks of training intervention. As shown in Table 3 and Figure 2, we found a few significant correlations between AA t0 concentrations and clinical parameters. Specifically, we could detect positive correlations between Cit concentrations and changes in fasting blood glucose as well as between Tyr concentrations and changes in systolic RR. However, when analyzing carnitine and AC concentrations, we found a range of significant correlations with changes in clinical parameters, specifically relating to inflammation, glucose, lipid metabolism, and blood pressure/cardiovascular disease. In particular, a positive correlation was found between free carnitine and ΔCRP, as well as for SC- and MC-ACs with ΔHbA1c, Δfasting glucose, and Δcholesterol. In addition, t0 concentrations of free and total carnitine negatively correlated with ΔdiastRR und ΔsysRR. These data indicate that baseline concentrations of carnitine and ACs might represent candidate biomarkers in the context of training adaptation in this cohort.

### 3.3. Individual Acute Response at t2

**Figure 3:**
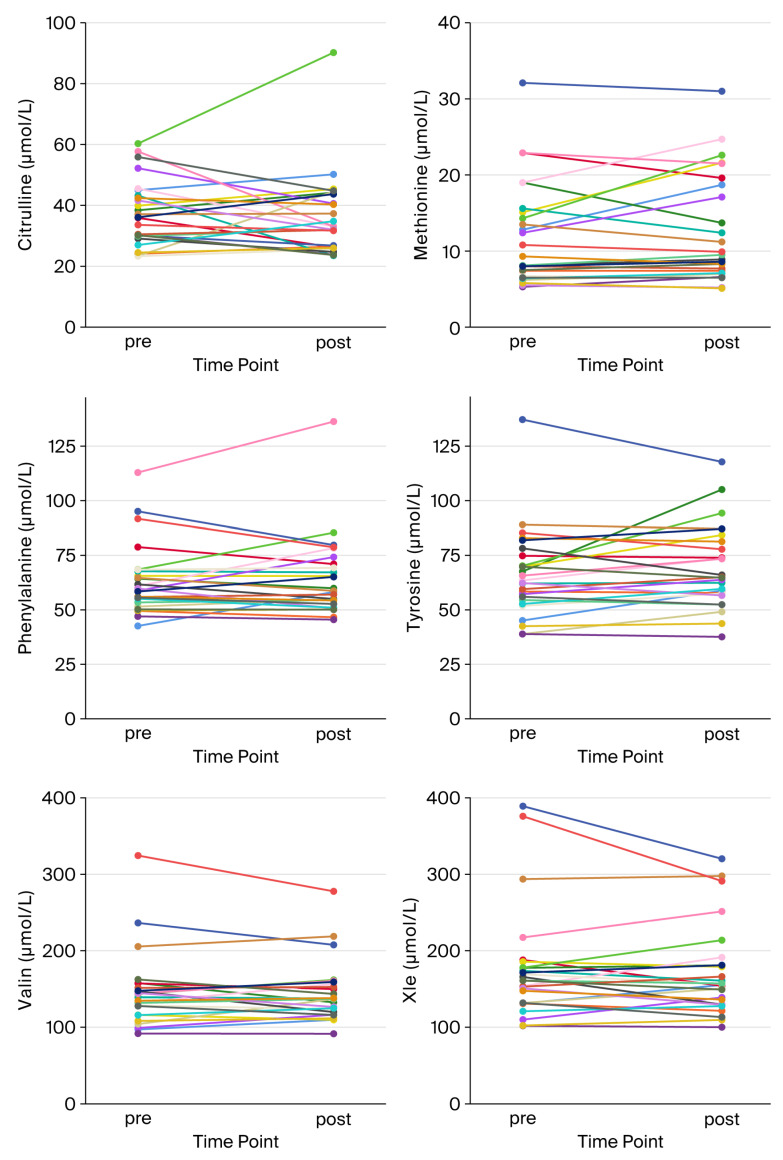

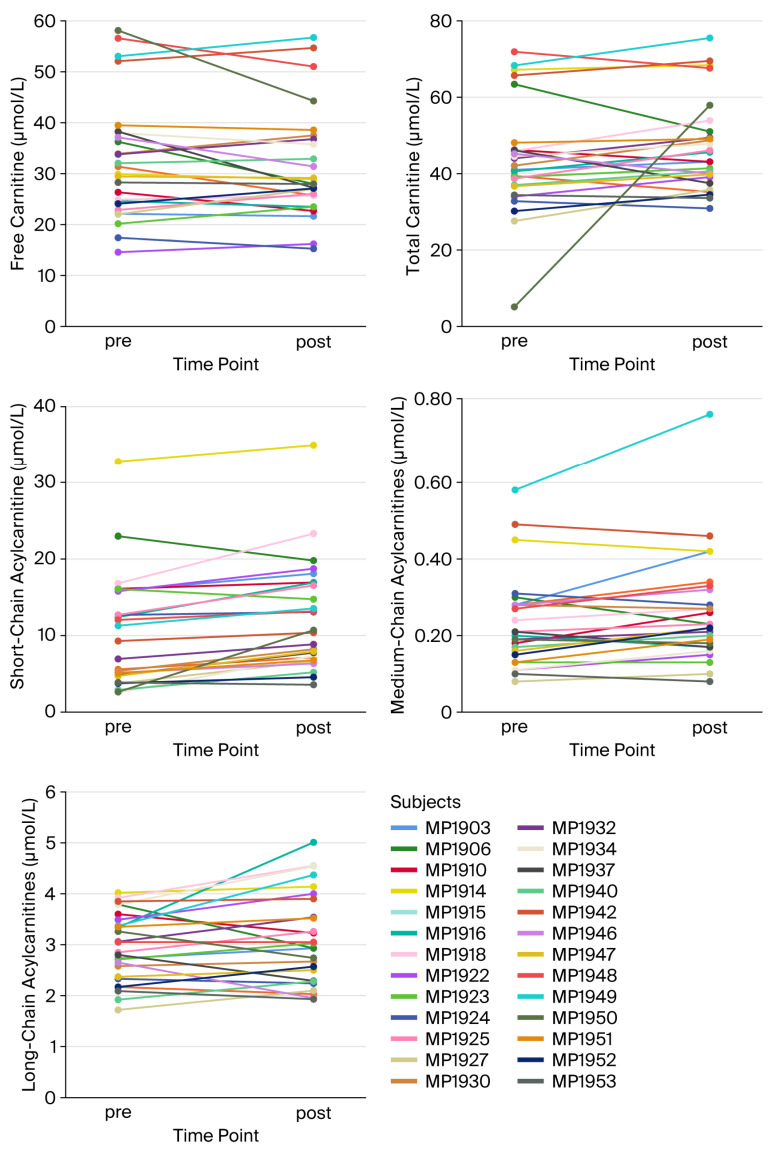
Spaghetti plots of individual acute responses at t2. Plots depict changes in individual metabolite concentrations during spiroergometry at t2 (acute response), as indicated.

In addition, we studied individual acute responses at t2 (n = 26). As shown in Table 4 and Figure 3, on average, significant increases were observed in SC- and MC-ACs in responses to exercise. All other metabolites did not show significant changes. However, large individual differences were still present, suggesting that metabolite patterns might be candidate markers in the context of exercise adaptation. When assessing potential correlations between metabolite mobilization and changes in physiological or clinical parameters between t0 and t2, we found a significant negative correlation between MC-AC acute response and changes in PPO (peak power output), indicating that subjects with lower rates of MC-AC mobilization might have higher gains in PPO (Table 5).

## 4. Discussion

Physical exercise is known to modulate AA plasma profiles, reflecting metabolic adaptations (for a review, see [20]). Our data indicate that the 24-week exercise-based lifestyle intervention program “MultiPill-Exercise” continuously upregulated concentrations of all tested AAs in circulation. In 2010, Kamaura et al. [21] carried out a similar study on subjects with metabolic syndrome undergoing a six-week lifestyle intervention program. Our results are in part consistent with theirs; specifically, they could also detect increased concentrations of Val, Tyr, and Cit after intervention. In contrast to our data, however, decreased concentrations of Leu and Phe were observed. These discrepancies might be due to differences in study population, exact nature of the intervention, and methodology, since Kamaura et al. [21] analyzed plasma samples, whereas in this study, dried whole-blood samples were employed. Interestingly, high AA concentrations in plasma can stimulate muscle protein synthesis, which might be a mechanism of training adaptation [22].

When considering AAs as potential biomarkers in the context of training adaptation, associations between AA patterns and changes in clinical and physiological parameters are particularly interesting. Thus, we correlated baseline AA concentrations with changes in such parameters between t0 and t1. Interestingly, we could detect positive correlations between Val, Xle, Tyr, and Phe and changes in clinical parameters such as BMI, waist circumference, systolic and diastolic blood pressure, fasting glucose, and LDL, as well as negative correlations with changes in HDL. In addition, we observed negative correlations between baseline Cit and Val concentrations and changes in VO_2_max, indicating that high concentrations of these AAs might be predictors of a weaker response to the lifestyle intervention program. These data confirm the results published by Kamaura et al. [21], who had also demonstrated several correlations between low baseline concentrations of various AAs and beneficial training outcomes.

By contrast, when analyzing AA mobilization in the context of acute exercise, almost no overall effect was observed, despite the partially huge inter-individual differences. A possible explanation might be that AA mobilization is less pronounced in subjects with low muscle mass, such as MultiPill-Exercise participants. In addition, timing might have played a role: Spiroergometry tests in MultiPill-Exercise subjects, which were organized as incremental tests until exhaustion, might, due to subjects’ comparatively low fitness, not have been long enough to detect significant AA mobilization.

When analyzing carnitine and AC levels throughout the intervention, we found a moderate increase in total carnitine and a non-significant decrease in free carnitine. Levels of all classes of ACs also increased, despite the fact that data for medium-chain ACs did not reach significance.

Interestingly, a negative correlation was found between baseline concentrations of free and total carnitine, and RR changes—both systolic and diastolic—suggesting that subjects with high levels of these metabolites at the start of this study had a higher cardiovascular benefit from the intervention.

Several studies have demonstrated distinct AC profiles in subjects with obesity, insulin resistance, and non-alcoholic fatty liver disease when compared with healthy controls [23,24]. In this study, we show that subjects with high concentrations of MC- and SC-ACs at inclusion had fewer positive effects with regard to fasting blood glucose and HbA1c amelioration when compared with subjects with lower baseline AC concentrations. In addition, high t0 concentrations of SC-ACs positively correlated with changes in cholesterol levels after 12 weeks. These data suggest that high AC concentrations might be negative predictors of training adaptation. The data presented by Carrard et al. [25] in a systematic review analyzing different metabolic markers with regard to their association with individual cardiorespiratory fitness support this assumption: The authors clearly demonstrated that high AC concentrations, independently of chain length, are negatively associated with cardiorespiratory fitness, supporting the hypothesis that the accumulation of circulating ACs reflects a state of compromised mitochondrial fatty acid oxidation, indicating poor metabolic health (for a review, see [12]). On the other hand, skeletal muscle ACs appear to be associated with a high degree of cardiovascular fitness, probably because they are linked to efficient fatty acid oxidation [26]. This is supported by results indicating that exercise can enhance mitochondrial fatty acid oxidation in skeletal muscle tissue [27].

Interestingly, we observed a significant increase in SC- and MC-ACs during acute exercise. A similar effect was previously described by Lehmann et al. [28], who demonstrated increases particularly in MC-ACs during 60 min runs, suggesting that these metabolites are released as byproducts/intermediates of partial β oxidation. Interestingly, a negative correlation was found between medium-chain AC mobilization and ΔPPO after 24 weeks, suggesting that subjects with a high degree of medium-chain AC mobilization might be characterized by poor training effects. By contrast, short- and long-chain AC mobilization were positively related to VO_2_max, suggesting that these subjects showed efficient training adaptation.

Finally, despite the fact that subjects were advised to only eat a light breakfast on testing days, with pretzels, apples, and cereal bars being offered to them, they were not in a fasted condition before spiroergometry, nor were their diets completely standardized, which might have influenced our results; exercise tolerance might have been lower in a fasted condition—especially in the previously mostly sedentary subjects of this study—and, thus, the probability of these subjects reaching their maximum power output might have been lower. Furthermore, for organizational reasons, spiroergometry is usually not carried out early in the morning, as it would be unethical to keep subjects in a fasted state until then. However, this heterogeneity was intended, to a degree, since fully standardizing diets for metabolomics diagnostics is usually not possible in clinical routine; i.e., useful biomarkers are required to function robustly in a real-world scenario of heterogeneous diets. Taken together, our data demonstrate that both AA and AC profiles are responsive to the lifestyle intervention program “MultiPill-Exercise” and might serve as biomarkers for clinical and physiological adaptation reactions in subjects with multimorbidity in the context of lifestyle intervention programs in the future.

## 5. Conclusions

Our data indicate that metabolomics patterns—specifically, those of AAs, C0/Cx, and ACs—might indeed be linked to changes in clinical and/or physiological data in the context of lifestyle intervention programs in subjects with multimorbidity. Further studies should focus on validation of the data in larger cohorts.

## 6. Study Limitations

The major limitation of our study is the limited sample size, requiring the need for further, confirmatory analyses: Due to restrictions imposed during the COVID-19 pandemic, complete sets of t0, t1, and t2 data were only available for n = 6 subjects of wave 1, all of whom were female. In addition, also due to the COVID-19 pandemic, the MultiPill-Exercise intervention design had to be substantially modified for subjects of wave 2, limiting comparability of the results obtained for the two waves. Furthermore, due to technical limitations, acute response data could only be collected at t2 (and not at t0), limiting the predictive value of these results. Finally, to maximize the number of subjects for whom data were available, correlations of baseline metabolomics data with changes in clinical and physiological parameters were carried out only for deltas between t0 and t1, i.e., at 12 weeks into the intervention. By contrast and aiming to maximize the number of analyzable data, acute response data were studied in the context of changes between t0 and t2 despite the fact that, as mentioned above, the second phase of the intervention had to be substantially modified for subjects of wave 2.

This was an exploratory, hypothesis-generating study; thus, no correction for multiple testing was introduced. Future studies should focus on validating metabolomics data in larger cohorts and potentially considering differences between male and female subjects, and different (co)morbidities. For this purpose, the hypotheses generated in this study are currently being tested and evaluated in a larger cohort of the “MultiPill-Exercise” main study, a larger, multicentric, randomized, and controlled trial which started in 2022 [29]. In addition, long-term longitudinal studies and multidisciplinary approaches—encompassing, for example, genetic or endocrinological data—would be interesting, as would mechanistic approaches, allowing for a deeper understanding of the metabolic pathways reflected in metabolomics patterns. Eventually, metabolomics screening in subjects with multimorbidity could constitute a tool in personalized sports medicine, allowing for the design of efficient, individual training regimens and lifestyle intervention programs. Finally, an important point is that in this study, only metabolites that are part of the standard newborn screening program were quantified. In the future, it will also be interesting to establish procedures allowing for the analysis of more specific metabolites—such as N-lactoylphenylalanine, which has recently been demonstrated to be an important modulator of exercise-induced weight loss in subjects with obesity (for a review, see [30])—from dried blood spots.

## Figures and Tables

**Table 1:** Metabolomics and abbreviations.

Abbreviations	Metabolites
Carnitine, C0	free/unconjugated carnitine
Cx	total carnitine (sum of free carnitine and all acylcarnitines)
C2	acetylcarnitine
C3-DC+C4-OH	malonylcarnitine (C3-DC) + 3-hydroxybutyrylcarnitine (C4-OH)
C3	propionylcarnitine (C3)
C4	butyrylcarnitine + isobutyrylcarnitine (C4)
C4-DC+C5-OH	methylmalonylcarnitine (C4-DC) + 3-hydroxyisovalerylcarnitine (C5-OH)
C5	isovalerylcarnitine + methylbutyrylcarnitine (C5)
C5-DC+C6-OH	glutarylcarnitine (C5-DC) + 3-hydroxyhexanoylcarnitine (C6-OH)
C6	hexanoylcarnitine (C6)
C6-DC	methylglutarylcarnitine (C6-DC)
C8	octanoylcarnitine (C8)
C10	decanoylcarnitine (C10)
C10:1	decenoylcarnitine (C10:1)
C12	dodecanoylcarnitine (C12)
C12:1	dodecenoylcarnitine (C12:1)
C14	tetradecanoylcarnitine (C14)
C14-OH	3-hydroxytetradecanoylcarnitine (C14-OH)
C14:1-OH	3-hydroxytetradecenoylcarnitine (C14:1-OH)
C14:1	tetradecenoylcarnitine (C14:1)
C14:2	tetradecadienoylcarnitine (C14:2)
C16	palmitoylcarnitine (C16)
C18	stearoylcarnitine (C18)
C18:1	oleoylcarnitine (C18:1)
C18:2	linoleoylcarnitine (C18:2)
C16-OH	3-hydroxypalmitoylcarnitine (C16-OH)
C18:1-OH	3-hydroxyoleoylcarnitine (C18:1-OH)
C18:2-OH	3-hydroxylinoleoylcarnitine (C18:2-OH)
SC-ACs	short-chain acylcarnitines (C2-C6)
MC-ACs	medium-chain acylcarnitines (C8-C12)
LC-ACs	long-chain acylcarnitines (>C12)
Cit	citrulline
Met	methionine
Phe	phenylalanine
Tyr	tyrosine
Val	valine
Xle	leucine + isoleucine

This table lists all the metabolites analyzed in this study and their abbreviations.

**Table 2:** Individual kinetics of metabolite concentrations (t0, t1, and t2).

		t	n	Min	Max	MD	ME	SD	Δ (t0–t1) p	Δ (t0–t2) p
amino acids	Cit (µmol/L)	t0	6	15.7	35.7	27.05	26.78	7.34	10.37 0.156	20.45 0.031 *
		t1	6	27.8	55.6	36.60	37.15	10.10		
		t2	6	30.1	60.3	47.70	47.23	11.56		
	Met (µmol/L)	t0	6	3.5	7.9	5.05	5.30	1.56	6.43 0.016 *	13.43 0.031 *
		t1	6	9.7	15.1	11.40	11.73	2.09		
		t2	6	12.4	32.1	15.35	18.73	7.47		
	Phe (µmol/L)	t0	6	34.8	56.6	44.65	44.83	9.63	14.10 0.031 *	33.27 0.013 *
		t1	6	42.6	93.8	54.15	58.93	19.04		
		t2	6	58.8	112.9	68.10	78.10	21.11		
	Tyr (µmol/L)	t0	6	39.9	53.5	47.95	47.83	5.36	13.07 0.109	29.20 0.031 *
		t1	6	50.2	70.7	62.70	60.90	7.91		
		t2	6	57.2	137.2	67.75	77.03	29.88		
	Val (µmol/L)	t0	6	73.1	129.2	86.25	91.12	20.87	22.60 0.016 *	59.17 0.031 *
		t1	6	82.0	134.0	116.55	113.72	18.58		
		t2	6	99.0	236.4	141.55	150.28	45.54		
	Xle (µmol/L)	t0	6	81.4	181.6	102.15	111.32	36.50	30.75 0.109	97.63 0.031 *
		t1	6	104.9	179.1	141.75	142.07	29.43		
		t2	6	109.9	389.1	181.80	208.95	94.96		
(acyl)carnitines	Carnitine (µmol/L)	t0	6	15.9	30.2	24.88	24.09	4.76	−0.43 0.844	−2.50 0.313
		t1	6	19.3	31.2	22.68	23.66	4.59		
		t2	6	14.6	29.9	21.53	21.59	5.44		
	Cx (µmol/L)	t0	6	20.2	37.1	31.20	30.28	5.54	5.75 0.047 *	11.78 0.031 *
		t1	6	30.7	42.4	35.15	36.03	5.24		
		t2	6	32.8	67.2	38.90	42.07	12.69		
	SC-ACs (µmol/L)	t0	6	2.9	5.7	3.93	4.01	1.09	5.88 0.016 *	13.07 0.031 *
		t1	6	8.6	11.7	9.47	9.89	1.27		
		t2	6	12.4	32.8	14.26	17.08	7.87		
	MC-ACs (µmol/L)	t0	6	0.1	0.3	0.15	0.17	0.06	0.01 0.484	0.07 0.313
		t1	6	0.1	0.2	0.20	0.18	0.06		
		t2	6	0.1	0.5	0.21	0.24	0.13		
	LC-ACs (µmol/L)	t0	6	1.3	2.6	2.08	2.02	0.45	0.27 0.031 *	1.10 0.031 *
		t1	6	1.7	3.4	2.17	2.30	0.59		
		t2	6	2.3	4.0	3.10	3.12	0.61		

Table shows concentrations of individual metabolites at the three different time points and their changes (D) between t0 and t1 and between t0 and t2. t: time point; n: number of subjects; min: minimum value; max: maximum value; MD: median; ME: mean; SD: standard deviation. *: *p* < 0.05.

**Table 3:** Baseline metabolomics (t0): correlation analysis.

Δphysiological/Clinical Parameters (t0–t1)		Statistics	AA Concentration (t0)						AC Concentration (t0)				
			Cit	Met	Phe	Tyr	Val	Xle	C0	Cx	SC-ACs	MC-ACs	LC-ACs
sports	ΔrelVO2max [mL/kgxmin]	correlation [ρ]	−0.624	−0.459	−0.532	−0.184	−0.716	−0.514	0.019	0.167	0.239	−0.278	0.184
		p (2-sided)	0.134	0.300	0.219	0.694	0.070	0.238	0.969	0.721	0.606	0.546	0.694
		n	7	7	7	7	7	7	7	7	7	7	7
	ΔPPO [W/kg]	correlation [ρ]	−0.144	0.180	−0.487	0.487	−0.054	0.018	−0.245	−0.373	0.108	−0.055	0.162
		p (2-sided)	0.758	0.699	0.268	0.268	0.908	0.969	0.596	0.41	0.818	0.908	0.728
		n	7	7	7	7	7	7	7	7	7	7	7
body weight	ΔBMI [kg/m^2^]	correlation [ρ]	−0.679	0.107	−0.036	0.214	−0.036	0.071	−0.577	−0.360	−0.179	−0.577	−0.321
		p (2-sided)	0.094	0.819	0.939	0.645	0.939	0.879	0.175	0.427	0.702	0.175	0.482
		n	7	7	7	7	7	7	7	7	7	7	7
inflammation	ΔIL6 [ng/L]	correlation[ρ]	0.643	−0.250	0.071	−0.143	0.036	−0.214	0.342	0.414	0.643	0.306	0.464
		p (2-sided)	0.119	0.589	0.879	0.760	0.939	0.645	0.452	0.355	0.119	0.504	0.294
		n	7	7	7	7	7	7	7	7	7	7	7
	ΔCRP [mg/dL]	correlation[ρ]	0.000	−0.071	0.536	−0.429	−0.071	0.143	0.775 *	0.595	−0.321	0.270	0.179
		p (2-sided)	1.000	0.879	0.215	0.337	0.879	0.76	0.041	0.159	0.482	0.558	0.702
		n	7	7	7	7	7	7	7	7	7	7	7
	Δfibrinogen [mg/dL]	correlation[ρ]	0.257	0.086	−0.029	0.429	0.029	0.086	0.261	0.174	0.429	0.116	0.486
		p (2-sided)	0.623	0.872	0.957	0.397	0.957	0.872	0.618	0.742	0.397	0.827	0.329
		n	6	6	6	6	6	6	6	6	6	6	6
diabetes	Δinsulin [pmol/L]	correlation[ρ]	0.000	0.107	−0.250	0.321	0.000	0.036	0.144	−0.072	0.143	0.090	0.429
		p (2-sided)	1.000	0.819	0.589	0.482	1.000	0.939	0.758	0.878	0.76	0.848	0.337
		n	7	7	7	7	7	7	7	7	7	7	7
	ΔHbA1c [%]	correlation[ρ]	0.356	−0.225	0.337	−0.019	0.206	−0.131	0.198	0.415	0.767 *	−0.076	0.636
		p (2-sided)	0.434	0.628	0.460	0.968	0.658	0.78	0.67	0.354	0.044	0.872	0.125
		n	7	7	7	7	7	7	7	7	7	7	7
	Δglucose [mg/dL]	correlation[ρ]	0.757 *	0.090	−0.180	0.000	−0.234	0.054	0.500	0.291	0.126	0.836 *	0.018
		p (2-sided)	0.049	0.848	0.699	1.000	0.613	0.908	0.253	0.527	0.788	0.019	0.969
		n	7	7	7	7	7	7	7	7	7	7	7
lipoprotein and fatty acid metabolism	Δcholesterol [mg/dL]	correlation[ρ]	0.214	−0.357	0.000	0.000	−0.286	−0.321	0.180	0.523	0.964 ***	−0.018	0.571
		p (2-sided)	0.645	0.432	1.000	1.000	0.535	0.482	0.699	0.229	<0.001	0.969	0.18
		n	7	7	7	7	7	7	7	7	7	7	7
	Δtriglycerides [mg/dL]	correlation[ρ]	−0.018	0.270	−0.450	0.468	−0.198	0.126	−0.073	−0.245	0.000	0.209	0.018
		p (2-sided)	0.969	0.558	0.31	0.289	0.67	0.788	0.877	0.596	1	0.653	0.969
		n	7	7	7	7	7	7	7	7	7	7	7
	ΔLDL [mg/dL]	correlation[ρ]	0.679	−0.214	−0.143	−0.071	0.000	−0.250	0.180	0.234	0.607	0.342	0.321
		p (2-sided)	0.094	0.645	0.76	0.879	1	0.589	0.699	0.613	0.148	0.452	0.482
		n	7	7	7	7	7	7	7	7	7	7	7
	ΔHDL [mg/dL]	correlation[ρ]	0.144	−0.108	0.360	−0.018	0.559	−0.036	−0.018	0.027	0.324	−0.309	0.505
		p (2-sided)	0.758	0.818	0.427	0.969	0.192	0.939	0.969	0.954	0.478	0.5	0.248
		n	7	7	7	7	7	7	7	7	7	7	7
cardiovascular parameters	ΔdiastRR [mmHg]	correlation[ρ]	0.072	0.414	−0.360	0.577	0.342	0.198	−0.927 **	−0.855 *	−0.090	−0.191	−0.541
		p (2-sided)	0.878	0.355	0.427	0.175	0.452	0.67	0.003	0.014	0.848	0.682	0.21
		n	7	7	7	7	7	7	7	7	7	7	7
	ΔsysRR [mmHg]	correlation[ρ]	−0.324	0.667	−0.036	0.757 *	0.631	0.523	−0.873 *	−0.964 ***	−0.450	−0.445	−0.396
		p (2-sided)	0.478	0.102	0.939	0.049	0.129	0.229	0.01	<0.001	0.31	0.317	0.379
		n	7	7	7	7	7	7	7	7	7	7	7
	Δresting HR [bpm]	correlation[ρ]	−0.074	−0.296	−0.111	−0.037	0.111	−0.296	0.187	0.112	0.408	−0.224	0.741
		p (2-sided)	0.875	0.518	0.812	0.937	0.812	0.518	0.688	0.811	0.364	0.629	0.057
		n	7	7	7	7	7	7	7	7	7	7	7

Correlation of baseline (t0) metabolite concentrations and changes in physiological and clinical parameters between t0 and t1, as indicated. *ρ*: correlation coefficient (Spearman). *: *p* < 0.05, **: *p* < 0.01, ***: *p* < 0.001.

**Table 4:** Acute response at t2.

		t2	n	Min	Max	MD	ME	SD	*p*
amino acids	Cit (µmol/L)	pre	26	23.3	60.3	36.00	37.20	10.76	
		post	26	23.5	90.2	32.60	36.03	13.70	
		Δ	26	−24.6	29.9	−0.85	−1.17	11.40	0.464
	Met (µmol/L)	pre	26	5.3	32.1	8.70	11.89	6.73	
		post	26	5.1	31.0	9.20	12.57	7.13	
		Δ	26	−5.3	8.3	0.00	0.67	3.20	0.579
	Phe (µmol/L)	pre	26	42.6	112.9	59.30	63.22	15.97	
		post	26	45.5	136.3	58.45	64.23	18.47	
		Δ	26	−15.4	23.4	−0.30	1.00	9.67	0.886
	Tyr (µmol/L)	pre	26	38.9	137.2	62.90	65.99	20.07	
		post	26	37.6	117.8	64.85	69.34	18.76	
		Δ	26	−19.4	37.5	0.75	3.35	11.32	0.210
	Val (µmol/L)	pre	26	91.7	324.5	139.35	145.65	48.15	
		post	26	91.4	277.7	137.15	144.50	39.17	
		Δ	26	−46.8	32.7	0.45	−1.14	18.37	0.920
	Xle (µmol/L)	pre	26	101.8	389.1	161.00	175.87	72.28	
		post	26	100.0	320.3	155.85	171.62	58.62	
		Δ	26	−84.8	37.5	−2.80	−4.26	29.30	0.745
(acyl-)carnitines	C0 (µmol/L)	pre	26	14.6	58.1	30.64	32.57	11.77	
		post	26	15.3	56.7	27.98	31.35	10.67	
		Δ	26	−13.8	4.9	−0.40	−1.22	4.76	0.437
	Cx (µmol/L)	pre	26	5.1	71.9	41.55	43.66	14.52	
		post	26	30.9	75.5	44.35	47.12	12.03	
		Δ	26	−12.4	52.8	3.20	3.46	11.35	0.070
	SC-ACs (µmol/L)	pre	26	2.6	32.8	8.12	10.16	7.29	
		post	26	3.6	35.0	10.55	12.36	7.06	
		Δ	26	−3.2	8.1	2.19	2.20	2.27	<0.001 **
	MC-ACs (µmol/L)	pre	26	0.1	0.6	0.21	0.23	0.12	
		post	26	0.1	0.8	0.23	0.26	0.14	
		Δ	26	−0.1	0.2	0.03	0.03	0.06	0.027 *
	LC-ACs (µmol/L)	pre	26	1.7	4.0	2.95	2.96	0.67	
		post	26	1.9	5.0	2.98	3.13	0.90	
		Δ	26	−0.9	1.7	0.15	0.17	0.54	0.147

This table shows the changes in concentrations of individual metabolites during spiroergometry at t2. pre: before spiroergometry; post: after spiroergometry; Δ: difference; n: number of subjects; min: minimum value; max: maximum value; MD: median; ME: mean; SD: standard deviation. *: *p* < 0.05, **: *p* < 0.01.

**Table 5:** Correlation between acute response and changes in physiological/clinical parameters.

Δphysiological/Clinical Parameters (t0–t2)		Statistics	Amino Acid Mobilization During Acute Bout of Exercise at t2						(Acyl-)carnitine Mobilization During Acute Bout of Exercise at t2				
			Cit	Met	Phe	Tyr	Val	Xle	C0	Cx	SC- ACs	MC-ACs	LC- ACs
sports	ΔrelVO2max [mL/kgxmin]	correlation [ρ]	−0.047	0.012	−0.002	−0.034	0.014	−0.087	−0.079	−0.040	0.230	−0.170	0.124
		p (2-sided)	0.819	0.955	0.993	0.867	0.945	0.672	0.703	0.846	0.258	0.407	0.547
		n	26	26	26	26	26	26	26	26	26	26	26
	ΔPPO [W/kg]	correlation [ρ]	0.024	−0.073	−0.170	−0.077	−0.121	−0.158	−0.001	−0.011	0.044	−0.480 *	0.113
		p (2-sided)	0.910	0.735	0.427	0.719	0.573	0.460	0.997	0.960	0.838	0.018	0.599
		n	24	24	24	24	24	24	24	24	24	24	24
body weight	ΔBMI [kg/m^2^]	correlation [ρ]	−0.090	−0.324	−0.080	0.015	−0.169	−0.032	0.017	−0.028	−0.266	−0.124	−0.240
		p (2-sided)	0.663	0.107	0.699	0.941	0.409	0.875	0.935	0.893	0.188	0.545	0.238
		n	26	26	26	26	26	26	26	26	26	26	26

Correlation of acute changes in metabolite concentrations during spiroergometry at t2 and changes in physiological and clinical parameters between t0 and t2, as indicated. *ρ*: correlation coefficient (Spearman). *: *p* < 0.05

## Data Availability

The original contributions presented in this study are included in the article. Further inquiries can be directed to the corresponding author(s).

## References

[1] Nicholson K., Makovski T.T., Nagyova I., van den Akker M., Stranges S. (2023). Strategies to improve health status among adults with multimorbidity: A scoping review. Maturitas.

[2] Schweda S., Munz B., Burgstahler C., Niess A.M., Roesel I., Sudeck G., Krauss I. (2022). Proof of Concept of a 6-Month Person-Oriented Exercise Intervention ‘MultiPill-Exercise’ among Patients at Risk of or with Multiple Chronic Diseases: Results of a One-Group Pilot Trial. Int. J. Environ. Res. Public Health.

[3] Sakaguchi C.A., Nieman D.C., Signini E.F., Abreu R.M., Catai A.M. (2019). Metabolomics-Based Studies Assessing Exercise-Induced Alterations of the Human Metabolome: A Systematic Review. Metabolites.

[4] Schranner D., Kastenmüller G., Schönfelder M., Römisch-Margl W., Wackerhage H. (2020). Metabolite Concentration Changes in Humans After a Bout of Exercise: A Systematic Review of Exercise Metabolomics Studies. Sports Med. Open.

[5] Tian Q., Corkum A.E., Moaddel R., Ferrucci L. (2021). Metabolomic profiles of being physically active and less sedentary: A critical review. Metabolomics.

[6] Khoramipour K., Sandbakk Ø., Keshteli A.H., Gaeini A.A., Wishart D.S., Chamari K. (2022). Metabolomics in Exercise and Sports: A Systematic Review. Sports Med..

[7] Flynn N.E., Shaw M.H., Becker J.T. (2020). Amino Acids in Health and Endocrine Function. Adv. Exp. Med. Biol..

[8] Hansen J.S., Zhao X., Irmler M., Liu X., Hoene M., Scheler M., Li Y., Beckers J., Hrabĕ de Angelis M., Häring H.U. (2015). Type 2 diabetes alters metabolic and transcriptional signatures of glucose and amino acid metabolism during exercise and recovery. Diabetologia.

[9] Rinaldo P., Cowan T.M., Matern D. (2008). Acylcarnitine profile analysis. Genet. Med..

[10] Adeva-Andany M.M., Calvo-Castro I., Fernández-Fernández C., Donapetry-García C., Pedre-Piñeiro A.M. (2017). Significance of l-carnitine for human health. IUBMB Life.

[11] Rutkowsky J.M., Knotts T.A., Ono-Moore K.D., McCoin C.S., Huang S., Schneider D., Singh S., Adams S.H., Hwang D.H. (2014). Acylcarnitines activate proinflammatory signaling pathways. Am. J. Physiol. Endocrinol. Metab..

[12] McCoin C.S., Knotts T.A., Adams S.H. (2015). Acylcarnitines--old actors auditioning for new roles in metabolic physiology. Nat. Rev. Endocrinol..

[13] Huffman K.M., Slentz C.A., Bateman L.A., Thompson D., Muehlbauer M.J., Bain J.R., Stevens R.D., Wenner B.R., Kraus V.B., Newgard C.B. (2011). Exercise-induced changes in metabolic intermediates, hormones, and inflammatory markers associated with improvements in insulin sensitivity. Diabetes Care.

[14] Huffman K.M., Koves T.R., Hubal M.J., Abouassi H., Beri N., Bateman L.A., Stevens R.D., Ilkayeva O.R., Hoffman E.P., Muoio D.M. (2014). Metabolite signatures of exercise training in human skeletal muscle relate to mitochondrial remodelling and cardiometabolic fitness. Diabetologia.

[15] Skogvold H.B., Rootwelt H., Reubsaet L., Elgstøen K.B.P., Wilson S.R. (2023). Dried blood spot analysis with liquid chromatography and mass spectrometry: Trends in clinical chemistry. J. Sep. Sci..

[16] Koal T., Deigner H.P. (2010). Challenges in mass spectrometry based targeted metabolomics. Curr. Mol. Med..

[17] la Marca G. (2014). Mass spectrometry in clinical chemistry: The case of newborn screening. J. Pharm. Biomed. Anal..

[18] (2017). Newborn Screening by Tandem Mass Spectrometry. 2nd Ed.

[19] Millington D.S. (2024). How mass spectrometry revolutionized newborn screening. J. Mass Spectrom. Adv. Clin. Lab..

[20] Kelly R.S., Kelly M.P., Kelly P. (2020). Metabolomics, physical activity, exercise and health: A review of the current evidence. Biochim. Biophys. Acta. Mol. Basis Dis..

[21] Kamaura M., Nishijima K., Takahashi M., Ando T., Mizushima S., Tochikubo O. (2010). Lifestyle modification in metabolic syndrome and associated changes in plasma amino acid profiles. Circ. J..

[22] Wolfe R.R., Miller S.L. (1999). Amino acid availability controls muscle protein metabolism. Diabetes Nutr. Metab..

[23] Mihalik S.J., Goodpaster B.H., Kelley D.E., Chace D.H., Vockley J., Toledo F.G., DeLany J.P. (2010). Increased levels of plasma acylcarnitines in obesity and type 2 diabetes and identification of a marker of glucolipotoxicity. Obesity.

[24] Zeljkovic A., Mihajlovic M., Vujcic S., Guzonjic A., Munjas J., Stefanovic A., Kotur-Stevuljevic J., Rizzo M., Bogavac-Stanojevic N., Gagic J. (2023). The Prospect of Genomic, Transcriptomic, Epigenetic and Metabolomic Biomarkers for The Personalized Prevention of Type 2 Diabetes and Cardiovascular Diseases. Curr. Vasc. Pharmacol..

[25] Carrard J., Guerini C., Appenzeller-Herzog C., Infanger D., Königstein K., Streese L., Hinrichs T., Hanssen H., Gallart-Ayala H., Ivanisevic J. (2022). The Metabolic Signature of Cardiorespiratory Fitness: A Systematic Review. Sports Med..

[26] Purdom T., Kravitz L., Dokladny K., Mermier C. (2018). Understanding the factors that effect maximal fat oxidation. J. Int. Soc. Sports Nutr..

[27] Lundsgaard A.M., Fritzen A.M., Kiens B. (2018). Molecular Regulation of Fatty Acid Oxidation in Skeletal Muscle during Aerobic Exercise. Trends Endocrinol. Metab..

[28] Lehmann R., Zhao X., Weigert C., Simon P., Fehrenbach E., Fritsche J., Machann J., Schick F., Wang J., Hoene M. (2010). Medium chain acylcarnitines dominate the metabolite pattern in humans under moderate intensity exercise and support lipid oxidation. PLoS ONE.

[29] Schweda S., Müller G., Munz B., Sudeck G., Martus P., Dierkes K., Krauss I. (2022). Implementation and evaluation of an individualized physical exercise promotion program in people with manifested risk factors for multimorbidity (MultiPill-Exercise): A study protocol for a pragmatic randomized controlled trial. BMC Public Health.

[30] Naja K., Hedaya L., Elashi A.A., Rizzo M., Elrayess M.A. (2025). N-Lactoyl Amino Acids: Emerging Biomarkers in Metabolism and Disease. Diabetes Metab. Res. Rev..

